# Mucosal carcinoma arising in a colon diverticulum: endoscopic submucosal dissection using the novel “clip-band-tent” traction method

**DOI:** 10.1055/a-2584-1414

**Published:** 2025-05-06

**Authors:** Felipe Ramos-Zabala, Marian García-Mayor, Alejandra Alzina-Pérez, Raúl José Díaz-Molina, Luis Moreno-Almazán

**Affiliations:** 1 181073 Servicio de Gastroenterología, Hospital Universitario HM Montepríncipe, HM Hospitales, Boadilla del Monte, Madrid, Spain; 2 16345 Universidad San Pablo-CEU, CEU Universities, Madrid, Spain

Endoscopic submucosal dissection (ESD) may be considered challenging for treating a colonic tumor involving a diverticulum 1 or having tattoo-induced fibrotic submucosal 2 . Here, we report a case of a laterally spreading tumor (LST) tattooed arising from a colonic diverticulum resected by saline immersion ESD ( Video 1 ). A 83-year-old woman with a previous left hemicolectomy for sigmoid adenocarcinoma was referred to our hospital for an organ-sparing approach. On endoscopic examination, in the ascending colon, a lesion with granular nodular mixed LST morphology was detected inside of a diverticulum which was completely involved ( Fig. 1 ). Surface pattern evaluation classified this lesion as JNET2A according to the Japan NBI Expert Team classification. We performed the technique with the following steps ( Fig. 2 ): circumferential mucosal incision using the ERBEJET hydrodissection system (Erbe), which allowed the diverticulum to be released from the tattoo-induced fibrotic wall ( Fig. 3 ); the “clip-band-tent” traction method was used as a variant of the clip-band technique 3 for the eversion of the diverticulum, stabilizing the submucosal layer view and providing adequate tension, which allowed better recognition of the dissection line ( Fig. 4 ); we easily identified the fibrotic submucosal layer using immersion in saline solution and the clip-flap traction method 4 , which enabled precise dissection using the T-type HybridKnife in probe mode 5 and a VIO 3 unit set at preciseSECT mode (Erbe) with a narrow safety margin. En bloc resection was achieved without any adverse events ( Fig. 5 ) and the diverticular orifice and mucosal defect were closed using resolution clips (Boston Scientific). The patient was discharged 24 hours after ESD. Histopathological examination showed a well-differentiated adenocarcinoma confined to the mucosal layer and free lateral and vertical resection margins.

**Fig. 1:**
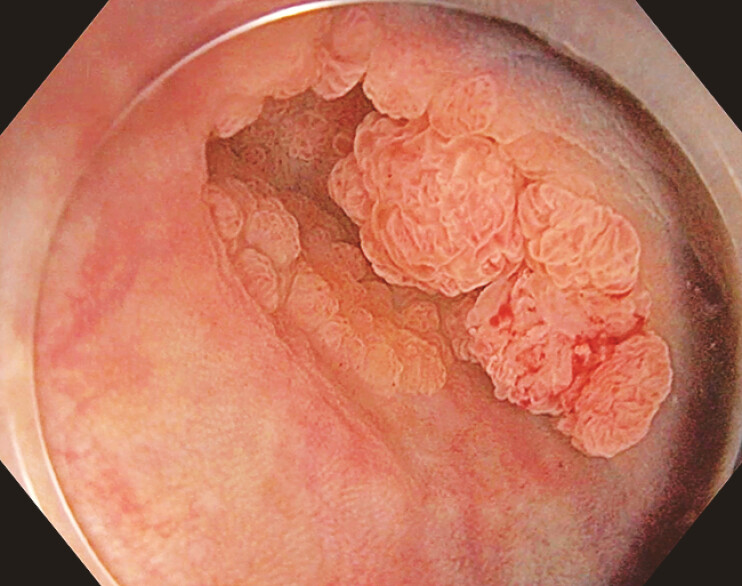
Endoscopic image showing a laterally spreading granular-type tumor in the ascending colon arising from the base of the diverticulum with evidence of a previous endoscopic tattoo around it. Surface pattern evaluation classified this lesion as JNET2A according to the Japan NBI Expert Team classification.

**Fig. 2:**
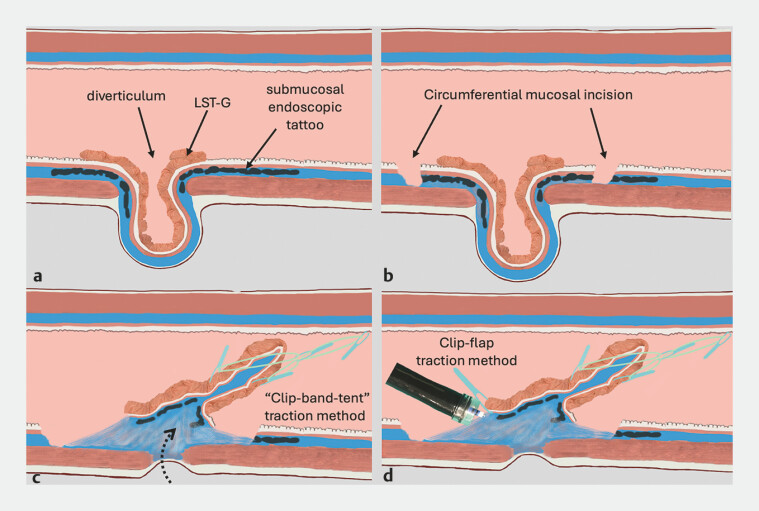
Graphical representation of endoscopic submucosal dissection with the “clip-band-tent” traction method for eversion of the diverticulum: **a** illustration of the laterally spreading granular-type tumor arising from the base of the diverticulum and endoscopic tattoo around it; **b** circumferential mucosal incision; **c** the “clip-band-tent” traction method is used as a variant of the clip-band technique for the eversion of diverticulum; **d** the clip-flap traction method is used facilitating submucosal layer visualization during saline immersion dissection.

**Fig. 3:**
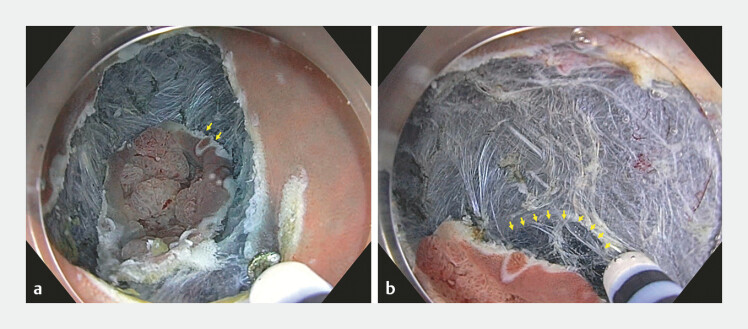
**a** Endoscopic image showing a circumferential mucosal incision T-type Hybridknife using an ERBEJET hydrodissection system, which allowed the diverticulum to be released from the tattoo-induced fibrotic wall. **b** The defect of the muscularis propria at the diverticulum is indicated with yellow arrows.

**Fig. 4:**
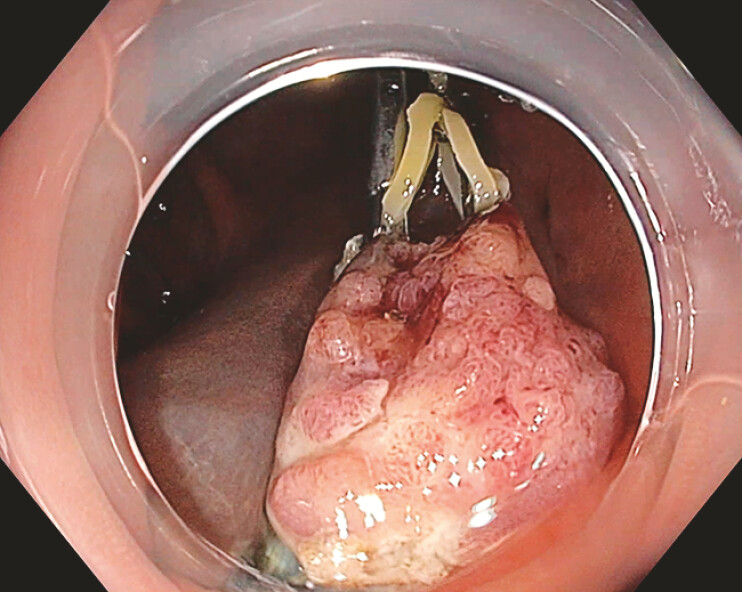
Endoscopic image showing an optimal eversion of the diverticulum by the “clip-band-tent” traction method.

**Fig. 5:**
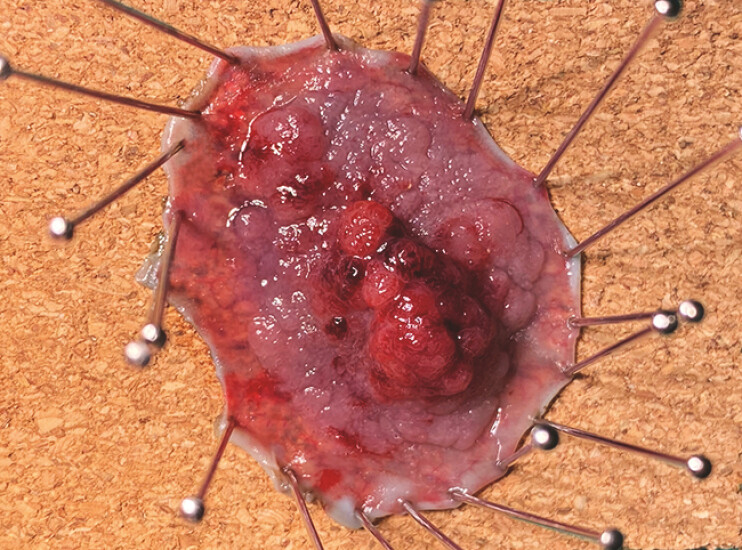
Macroscopic appearance of the resected specimen.

Large superficial tumor of the colon involving a diverticulum removed by endoscopic submucosal dissection using the novel “clip-band-tent” traction method and T-type Hybridknife used in probe mode.Video 1

The “clip-band-tent” traction method could be a promising variant of traction-assisted ESD for lesions involving a diverticulum as it significantly facilitates the precision of the technique.

Endoscopy_UCTN_Code_TTT_1AQ_2AD_3AD

## Notes

Endoscopy E-Videos https://eref.thieme.de/e-videos
*E-Videos* is an open access online section of the journal *Endoscopy* , reporting on interesting cases and new techniques in gastroenterological endoscopy. All papers include a high-quality video and are published with a Creative Commons CC-BY license. Endoscopy E-Videos qualify for HINARI discounts and waivers and eligibility is automatically checked during the submission process. We grant 100% waivers to articles whose corresponding authors are based in Group A countries and 50% waivers to those who are based in Group B countries as classified by Research4Life (see: https://www.research4life.org/access/eligibility/ ). This section has its own submission website at https://mc.manuscriptcentral.com/e-videos .
